# Modification of Processability and Shear-Induced Crystallization of Poly(lactic acid)

**DOI:** 10.3390/polym16243487

**Published:** 2024-12-14

**Authors:** Ruiqi Feng, Daisuke Kugimoto, Masayuki Yamaguchi

**Affiliations:** 1 Japan Advanced Institute of Science and Technology, Graduated School of Advanced Science and Technology, Asahidai, Nomi 923-1292, Ishikawa, Japan; mizuki_7@jaist.ac.jp; 2 Rolymer Materials Research Laboratory, Tosoh Corporation, 1-8 Kasumi, Yokkaichi 510-8540, Mie, Japan; daisuke-kugimoto-tv@tosoh.co.jp

**Keywords:** poly(lactic acid), polymer blend, modification

## Abstract

We studied the rheological properties under both shear and elongational flow and crystallization behaviors after shear history for binary blends of poly(lactic acid) (PLA) and ethylene–vinyl acetate copolymer (EVA) with a slightly lower shear viscosity. EVA was immiscible with PLA and dispersed in droplets in the blend. The addition of EVA significantly reduced the shear viscosity, which is attributed to the interfacial slippage between PLA and EVA. In contrast, under elongational flow, the addition of EVA provided strain hardening in the transient elongational viscosity. Consequently, the degree of neck-in behavior in T-die extrusion, i.e., a decrease in the film width, was reduced with the high orientation of the PLA chains. Furthermore, it was found that the addition of EVA accelerated the shear-induced crystallization of PLA, although EVA showed no nucleating ability without a flow field. Because the EVA addition can improve the mechanical toughness, this modification technique is attractive for various industrial applications of PLA.

## 1. Introduction

Poly(lactic acid) (PLA) stands out among polymers owing to its biocompatibility and availability from renewable agricultural resources [1,2,3,4,5,6]. However, despite these advantages, the application spectrum of PLA is constrained by its poor mechanical properties and processability, including brittleness and weak melt elasticity. Furthermore, slow crystallization impedes its processing efficiency. Recent studies have shown promising approaches to overcoming these challenges by modifying using blend techniques. Among them, the addition of ethylene–vinyl acetate copolymer (EVA) is advantageous for industrial processing of PLA [7,8,9,10,11,12,13] because it is a relatively simple method with good cost performance.

EVA is widely recognized to show good flexibility and impact resistance owing to its low crystallinity [14]. Moreover, it shows unique rheological properties—such as strain hardening in the transient elongational viscosity due to the presence of long-chain branches [15,16]—which are responsible for its good processability in various operations. Commercially available EVA is basically immiscible with PLA in the whole blend ratios and at any temperature [7,8,9,10,11,12,13]. The interfacial tension with PLA decreases with increasing the vinyl acetate content [8]. In previous papers [7,8,9,12], EVA with a large amount of vinyl acetate contents successfully improved the impact strength of PLA. For example, the EVA dispersion leads to shear yielding deformation, i.e., ductile deformation, when the ligament thickness is less than 2 μm [8]. Furthermore, EVA improved the processability during the production of a tubular-blown film [11]. Although the processability of PLA to produce a tubular-blown film is known to be improved by introducing chemical reactions [17,18], this often leads to the “fish-eye” problem. Therefore, the simple addition of EVA is an attractive approach. However, the processability of T-die extrusion has not been studied yet, although it is another important film production method. Since a high-quality film having homogeneous thickness is usually produced by T-die extrusion, it should be evaluated.

In this study, processability in T-die extrusion is first investigated, focusing on width reduction (so-called neck-in). Moreover, this study aims to further explore the role of EVA in the crystallization behavior of PLA under flow conditions. Although flow-induced crystallization has been a hot topic in polymer science for two decades, it has not been studied using an immiscible blend of PLA and rubbery materials. The findings could significantly influence the broader application of PLA as a biodegradable polymer.

## 2. Materials and Methods

### 2.1. Materials and Sample Preparation

Commercially available polymers, including poly(lactic acid) (PLA; Ingeo 4032D; NatureWorks, Minnetonka, MN, USA) and ethylene–vinyl acetate copolymer (EVA; Ultrathene 640; Tosoh, Tokyo, Japan) were used.

The L-lactide content of PLA was 98.5%, and its melting point was 167 °C. The density at 23 °C was 1240 kg m^−3^. The number- and weight-average molecular weights were 1.0 × 10^5^ and 1.8 × 10^5^ Da, respectively, as a polystyrene standard. The melt flow rate at 210 °C under 21.17 N was 7 g/10 min.

The vinyl acetate content in EVA was 25 wt%. The melting point was approximately 75 °C, and the density at 23 °C was 948 kg m^−3^. The number- and weight-average molecular weights were 4.2 × 10^4^ and 1.6 × 10^5^ Da, respectively, as a polyethylene standard. The melt flow rate at 190 °C under 21.17 N was 2.8 g/10 min.

After vacuum drying at 80 °C for 3 h, PLA and EVA were melt-mixed using a 30 cc internal batch mixer (Labo Plastmill 10M-100; Toyo Seiki Seisakusho, Tokyo, Japan) at 180 °C for 5 min. The blade rotation speed was 30 rpm, which provided a shear rate of 91 s^−1^ between the blades and the inner wall. The blend ratio of PLA/EVA by weight was 70/30. The same processing history was applied to pure PLA. After removing from the mixer, the blends were compression-molded into flat sheets with various thicknesses at 180 °C, then quenched at 25 °C.

T-die extrusion was performed using a single-screw extruder with a conical screw (Lumcr-50; Labtech Engineering, Samutprakarn, Thailand). The diameter at the bottom position of the screw was 18 mm, and that at the top was 8 mm. The length-to-diameter (top part) ratio was 30. Both PLA and EVA pellets were mixed in the solid state and fed into the extruder with a T-shaped die of 50 mm in width and 0.25 mm in die clearance. The output rate was 0.36 kg h^−1^ for both pure PLA and PLA/EVA (70/30). The die temperature was controlled at 200 °C, and the extruded film was stretched at 4 m min^−1^ using winding rolls. The chill roll was controlled at 50 °C, and the film thickness was approximately 30 μm. The shear rate on the die wall, ${\dot{\gamma }}_{wall}$, was calculated to be 590 s^−1^, using the following equation [19], assuming that the melt density was 1150 kg m^−3^ [20].

(1) $${\dot{\gamma }}_{wall}=\frac{6Q}{H^{2}W}$$

where *Q* is the volume flow rate, *H* is the die clearance, and *W* is the die width.

### 2.2. Measurements

The angular frequency, *ω*, dependence of the oscillatory shear moduli was examined using a cone-and-plate rheometer (AR2000ex; TA Instruments, New Castle, DE, USA). The cone angle was 4°, and the diameter was 25 mm. Measurements were conducted at 180 °C in an angular frequency range of 628.3–0.01 rad s^−1^. The transient elongational viscosity was determined using the rheometer with an extensional viscosity accessory (SER2-G; Xpansion Instruments, Tallmadge, OH, USA) operating under various Hencky strain rates ranging from 0.1 to 3.2 s^−1^ at 180 °C. Rectangular specimens (10 mm wide × 15 mm long × 1 mm thick) were cut out of the compression-molded sheets and used for the measurements.

The capillary extrusion was performed at 180 °C to evaluate the shear viscosity under pressure flow. A capillary rheometer (140-SAS-2002; Yasuda Seiki Seisakusyo, Nishinomiya, Japan) with a circular die was used. The length and diameter of the die were 40 mm and 1 mm, respectively, with an entrance angle of 2π. Neither Bagley nor Rabinowitsch corrections were applied. The extruded strands were collected to evaluate the flow instability.

Thermal properties were evaluated by differential scanning calorimetry (DSC; DSC8500; PerkinElmer, Waltham, MA, USA) under a purified nitrogen environment. Approximately 5.5 mg of the sample was enclosed in an aluminum pan and heated from 30 °C to 180 °C at a rate of 30 °C min^−1^. It was then cooled at 2 and 30 °C min^−1^ to 30 °C.

The blend morphology was investigated by scanning electron microscopy (SEM; TM3030; Hitachi, Tokyo, Japan). Prior to the observation, the cryogenically fractured surface of the compression-molded sheet was sputter-coated with Pt–Pd.

The temperature dependence of the dynamic tensile moduli was measured at 10 Hz using a dynamic mechanical analyzer (Rheogel E4000; UBM, Muko, Japan) in the temperature range from −80 °C to 180 °C. The heating rate was 2 °C min^−1^. Rectangular samples (5 mm wide × 20 mm long × 1 mm thick) cut from the compression-molded sheets were used.

The crystallization behavior of film samples with and without shear histories was evaluated by light transmittance under crossed polarizers through a parallel plate shear stage made of quartz (CSS450; Linkam Scientific Instruments, Surrey, UK). The parallel plate stage with a 2.8-mm-diameter window was contained in a temperature-controlled chamber, which was set in a polarized optical microscope (POM; Leica DMLP; Leica Microsystems, Wetzlar, Germany). The angles between the flow direction and both the polarizer and analyzer were 45°. The gap between plates was 100 µm. The shear was applied during the cooling process at various shear rates at the window position (7.5 mm from the center) by rotation of the bottom plate. The light intensity was collected using a photodetector (PM16-121; Thorlabs, Newton, MA, USA), which was installed instead of one of the eyepieces after passing through a 633-nm bandpass filter. A camera was set up on the other eyepiece to investigate the morphology using white light. The details and some experimental results obtained using this apparatus were reported in our previous papers [21,22]. The light transmittance, defined as the depolarized light intensity (DLI), was calculated using the following equation:

(2) $$DLI(\%)=\frac{I_{0}-I_{X}}{I_{//}-I_{X}}\times 100$$

where *I*_0_ is the light intensity passing through a sample under crossed polarizers, and *I_X_* and *I_//_* are the light intensities without a sample under crossed and parallel polarizers, respectively.

The molecular orientation of the T-die extruded films was evaluated using the POM under crossed polarizers. Optical retardation values were measured five times using a Berek compensator, and the average values were calculated.

## 3. Results and Discussion

### 3.1. Structure of Blend Sample

**Figure 1:**
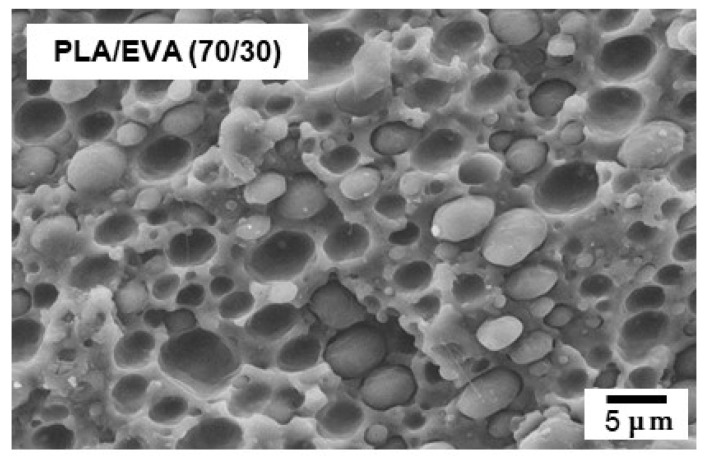
SEM image of the fractured surface of the PLA/EVA (70/30) film prepared by compression molding.

An SEM image of the blend sample prepared by compression molding is shown in Figure 1. A phase-separated structure was clearly observed, demonstrating that the EVA sample used in this study is immiscible with PLA. The average diameter of the spherical EVA particles was approximately 4.9 μm, and its standard deviation was 3.0 μm. The miscibility between the samples used in this study was previously studied with various blend ratios and found to be immiscible in the whole blend ratios at processing temperatures [8]. From the viewpoint of mechanical toughness, smaller EVA particles are better, as revealed by the previous research [8]. When using EVA with a high vinyl acetate content, the dispersion size is reduced with a low interfacial tension. However, the dispersion size does not affect the non-linear rheological properties [11].

**Figure 2:**
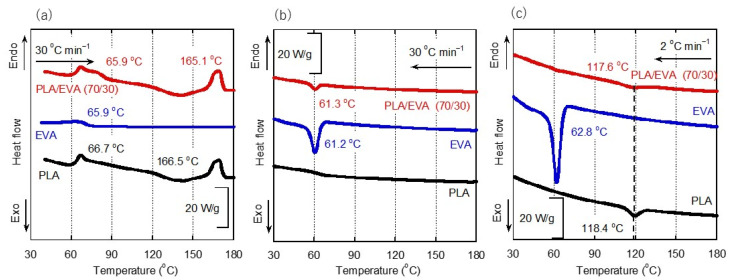
Differential scanning calorimetry (DSC) heating and cooling curves for the sample films prepared by compression molding. (**a**) Heating at 30 °C min^−1^, (**b**) cooling at 30 °C min^−1^, and (**c**) cooling at 2 °C min^−1^.

Figure 2 shows the DSC heating and cooling curves of the compression-molded sheets of PLA, EVA, and PLA/EVA (70/30). During the first heating run, a stepwise change was clearly detected at around 66 °C for both PLA and PLA/EVA (70/30) samples, ascribed to the glass-to-rubber transition. This indicates that the compression-molded sheets had no/few crystals, which is a typical observation for PLA products obtained by the quench process [23]. The melting point of pure EVA was around 66 °C. For the blend, a weak melting peak was detected after the glass transition, which is attributed to the melting of EVA. During heating, cold crystallization was detected from 120 to 160 °C before the melting of PLA at around 165 °C.

Crystallization behavior was evaluated at two cooling rates. Under rapid cooling conditions, i.e., 30 °C min^−1^, both PLA and PLA/EVA (70/30) exhibited no significant PLA crystallization peaks. The peak at around 50 °C in the blend is ascribed to EVA crystallization, which was, of course, detected in the cooling curve of pure EVA. In contrast, at a slow cooling rate of 2 °C min^−1^, PLA crystallization peaks were detected at around 118 °C for both samples. Moreover, the figure demonstrates that EVA showed no nucleating activity for PLA.

The enthalpies of cold crystallization and melting at the first heating and crystallization at the slow cooling rate (2 °C min^−1^) were summarized in Table 1.

**Figure 3:**
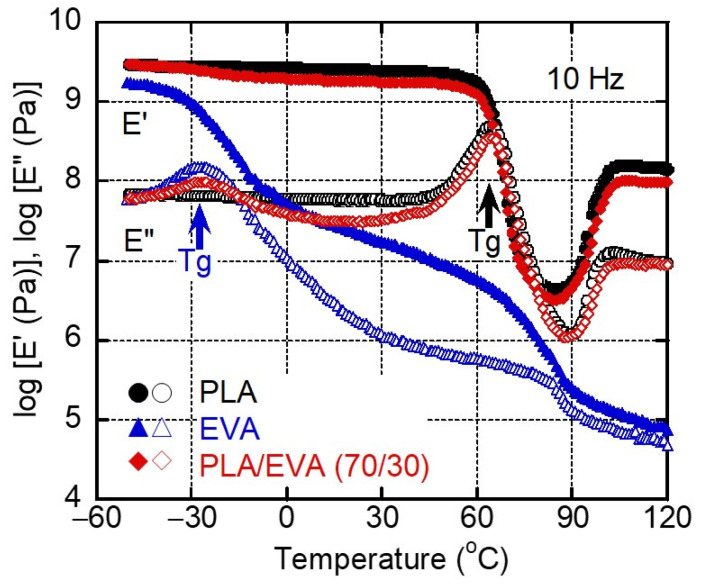
Temperature dependence of (closed symbols) tensile storage modulus *E′* and (open symbols) loss modulus *E″* at 10 Hz for (circles) PLA, (triangles) EVA, and (diamonds) PLA/EVA (70/30).

Figure 3 shows the temperature dependence of the dynamic tensile moduli, that is, the tensile storage modulus *E′* and loss modulus *E″*, for the compression-molded films. Both PLA and the blend showed a rapid decrease in *E′* above 60 °C, and both moduli increased at around 85 °C due to cold crystallization during heating. EVA did not affect the cold crystallization behavior for PLA, which corresponded with the DSC results. The *E′* values of EVA at room temperature were much lower than those of PLA and decreased rapidly around its melting point, i.e., ca. 75 °C. The peak temperature in the *E″* curve indicated the glass transition temperature *T_g_*, which was approximately 65 °C for PLA and −28 °C for EVA. In the blend, both the PLA and EVA phases displayed separate transitions with no shift in the peak temperatures of *E″*, i.e., *T_g_*. This result demonstrates that there was no mutual dissolution between the two polymers, as revealed before [7,8,9,12].

### 3.2. Rheological Properties

**Figure 4:**
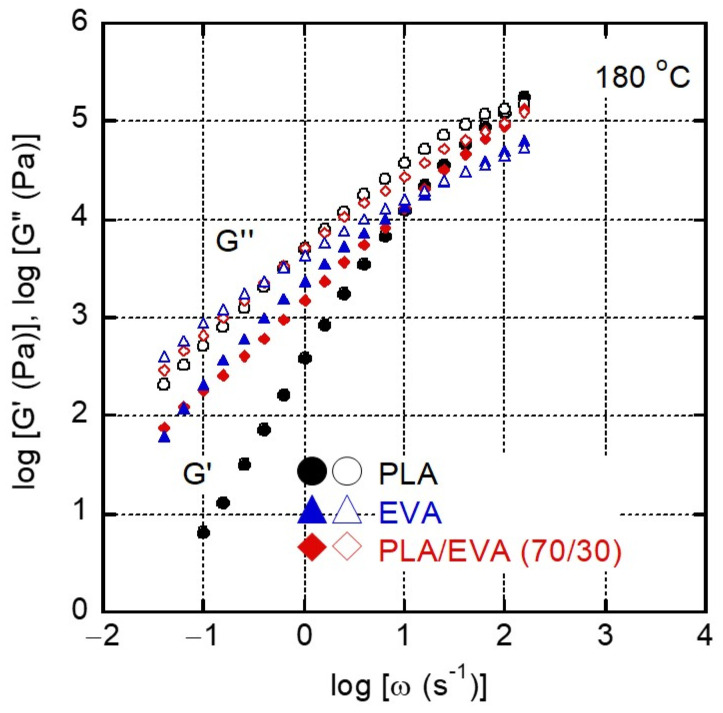
Angular frequency dependence of (closed symbols) shear storage modulus *G*’ and (open symbols) loss modulus *G*″ at 180 °C for (circles) PLA, (triangles) EVA, and (diamonds) PLA/EVA (70/30).

Figure 4 shows the angular frequency *ω* dependence of the shear storage modulus *G*’ and loss modulus *G″* for PLA, EVA, and PLA/EVA (70/30) at 180 °C. Pure PLA exhibited typical behavior in the rheological terminal/flow region, where *G′* and *G″* were proportional to *ω*^2^ and *ω*, respectively. For EVA, the slope of *G′* was markedly less than 2, demonstrating the existence of a long relaxation time mechanism. The broad molecular weight distribution with long-chain branches is expected to be responsible for the prolonged relaxation. The zero-shear viscosity *η*_0_, defined by the following relationship, was calculated as follows: 5.2 × 10^3^ Pa s for PLA, 9.5 × 10^3^ Pa s for EVA, and 7.2 × 10^3^ Pa s for PLA/EVA (70/30).

(3) $$\eta_{0}=\underset{\omega \to 0}{\lim}\frac{G\prime \prime }{\omega }$$

The addition of 30% EVA to PLA significantly altered the rheological properties, with both *G*’ and *G*” increasing markedly across the frequency spectrum, especially in the low-frequency region, even though PLA was the continuous phase. This suggests that EVA dispersions provided a long relaxation time owing to the interfacial tension. At high frequencies, in contrast, the EVA addition did not strongly affect the viscoelastic properties. The moduli in this region were determined by the entanglement couplings. Because PLA was the continuous phase, this is a reasonable result. Based on the Cox–Merz empirical rule, the result indicated that EVA barely affects the shear viscosity, which will be further discussed later.

**Figure 5:**
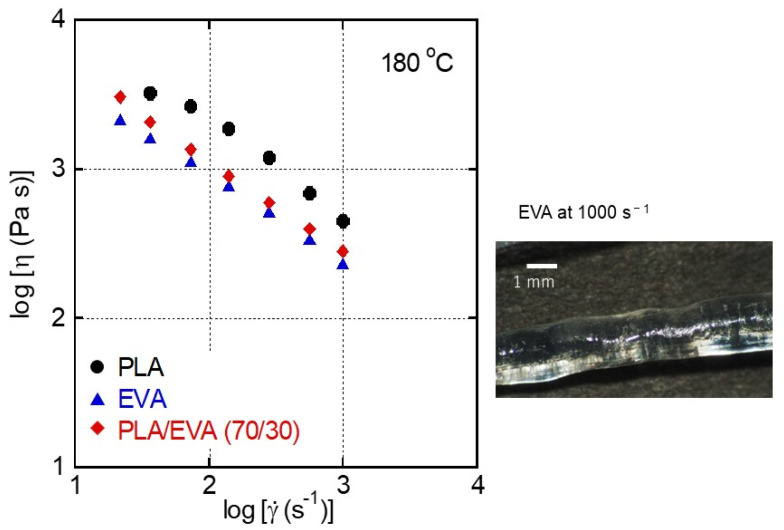
Flow curves evaluated by the capillary rheometer at 180 °C for (circles) PLA, (triangles) EVA, and (diamonds) PLA/EVA (70/30). The photograph of an EVA strand extruded at 1000 s^−1^ is shown on the right.

Figure 5 shows the flow curves, i.e., steady-state shear viscosity *η* plotted as a function of shear rate $\dot{\gamma }$, for PLA, EVA, and PLA/EVA (70/30), evaluated using a capillary rheometer. Both the shear viscosity and shear rate are the values at the die wall. No flow instability was detected for the samples, except for the gross volumetric melt fracture of EVA at the highest shear rate, i.e., 1000 s^−1^, as shown in the right figure. This is a typical flow instability for a long-chain branched polymer and originated from high elongational stress at die entrance [24].

The shear viscosities of EVA were lower than those of PLA in the wide shear rate range. Moreover, it should be noted that the blend showed similar viscosities to EVA, although PLA was the continuous phase. As mentioned previously, the oscillatory moduli in the high-frequency region of the blend were almost similar to those of pure PLA (Figure 4). These results demonstrate that the steady-state shear viscosity cannot be predicted by the linear viscoelastic properties. In other words, the addition of EVA unexpectedly reduced the shear viscosity of PLA to a great extent. This is advantageous in processing techniques, including injection molding. Recently, the importance of interfacial slippage at the phase boundary to shear viscosity under pressure flow was revealed for immiscible polymer blends with a dispersed phase with low viscosity [25,26,27]. It was reported that when a dispersion with low viscosity shows high interfacial tension with the continuous phase, the apparent shear viscosity decreases significantly because of the interfacial slippage. Consequently, the spiral flow length increases at injection molding [25], and the warpage of the injection-molded product is reduced [27]. The interfacial tension between the PLA and EVA used in this study was reported to be 2.4 mN m^−1^ [8]. Therefore, the interfacial thickness is very thin with no/few entanglement couplings. This is the origin of the interfacial slippage with a large interfacial area given by the deformation of dispersions.

**Figure 6:**
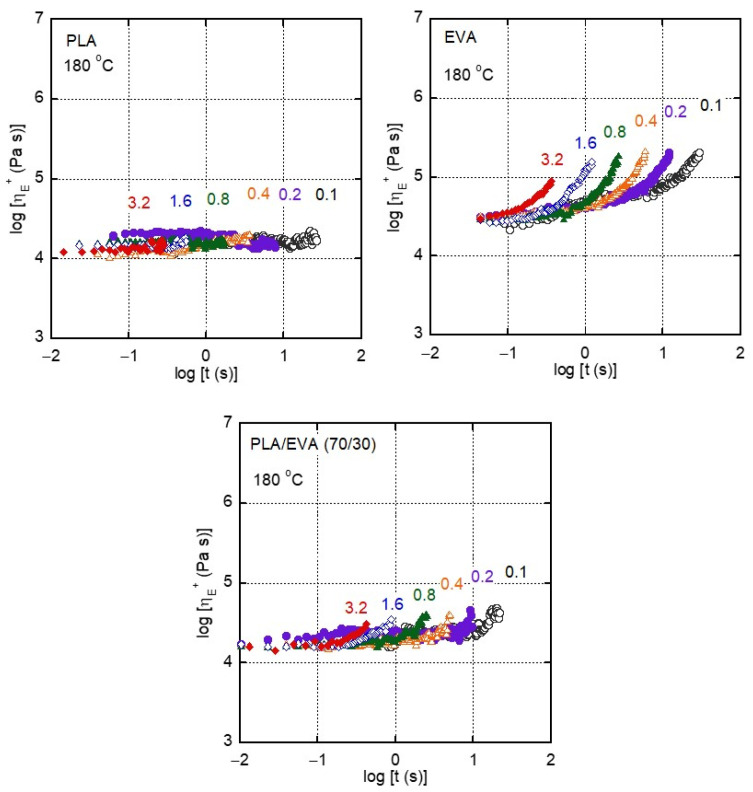
Elongational viscosity *η_E_*^+^ growth curves for PLA, EVA, and PLA/EVA (70/30) at 180 °C. The numerals in the figure represent the strain rates (s^−1^).

The transient elongational viscosity at 180 °C is shown in Figure 6. No strain hardening was detected at any strain rate for PLA. In contrast, EVA showed pronounced strain hardening, even at low strain rates, e.g., 0.1 s^−1^ and 0.2 s^−1^, which is thought to result from the long-chain branch structure similar to low-density polyethylene (LDPE) produced by radical polymerization at high pressure [24,28]. It should be noted that the blend clearly showed strain hardening even though EVA was in the dispersed phase. A comparable phenomenon has been reported for blends of polypropylene (PP) and LDPE [28,29,30]. According to them, dispersed LDPE droplets deformed along with the continuous phase, PP, during elongational flow. Eventually, the deformation of the LDPE droplets was negligible because of the stress increase, i.e., strain hardening in the elongational viscosity. As a result, the continuous PP must be significantly deformed, i.e., excess deformation in the PP phase. Such localized deformation increases the overall viscosity, as theoretically derived by Batchelor, known as the slender body theory [31]. In fact, the elongational viscosity growth curve was successfully predicted for PP/LDPE [30]. The same mechanism applies to this blend system.

It should be noted that the addition of EVA reduced the shear viscosity and provided the strain hardening in the elongational viscosity. This is expected to be advantageous for various processing operations, including T-die extrusion.

We also conducted T-die extrusion using PLA and PLA/EVA (70/30). For cast film processing, the neck-in phenomenon, i.e., width reduction of an extruded film, is important because it affects the production yield [32]. Therefore, the film width was evaluated using the same processing conditions, i.e., output rate and draw ratio. As can be seen in Table 2, the film width of pure PLA was 28.2 mm, and that of the blend was 30.0 mm. This demonstrates that the addition of EVA reduced the neck-in level even though the shear viscosity decreased. The neck-in level is known to have a close relationship with strain hardening in the transient elongational viscosity [33,34,35]. Because the blend showed strain hardening, as shown in Figure 6, the film width increased.

**Figure 7:**
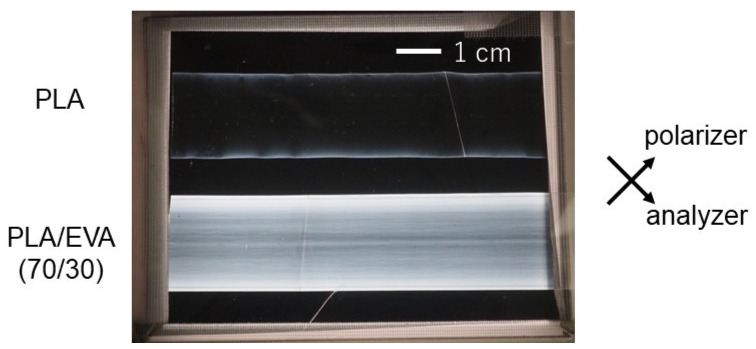
Extruded films under crossed polarizers.

Figure 7 shows the extruded films inserted between crossed polarizers. It is clear that the addition of EVA led to a bright image. Because the phase-separated structure leads to light scattering, which affects the light transmittance, we also evaluated the optical retardation using a Berek compensator. The results are summarized in Table 2. The PLA film had almost no orientation; however, the PLA/EVA film showed high birefringence. Considering the intrinsic birefringence of PLA is 0.03 [36], the Hermans orientation function for the blend film was estimated to be 0.045. Although the crystallinity was not enhanced by the addition of EVA under these processing conditions—which was confirmed by X-ray diffraction and DSC measurements—the chain orientation of PLA was enhanced. The excess strain in the continuous PLA phase applied in the air gap, which is the origin of the strain hardening in the transient elongational viscosity, is thought to result in the chain orientation.

### 3.3. Shear-Induced Crystallization

**Figure 8:**
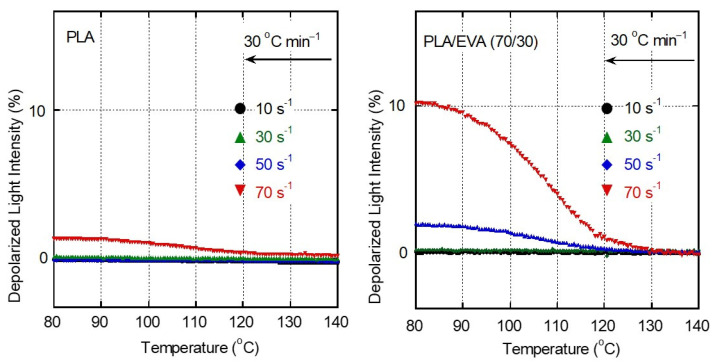
Depolarized light intensity (DLI) growth curves for PLA and the blend obtained during cooling at 30 °C min^−1^. The samples had a shear history from 190 to 160 °C at various shear rates.

As shown in Figure 2, PLA was not crystallized at a cooling rate of 30 °C min^−1^ without a flow field. Furthermore, the DSC measurements showed that the addition of EVA had little effect on the PLA crystallization. Such behavior was also detected by the light transmittance measurements under crossed polarizers, i.e., the depolarized light intensity (DLI), without exposure to shear flow. When a shear history was applied during cooling, however, PLA crystallization was detected, as shown in Figure 8. In the case of PLA, the DLI values increased slightly after a shear flow at 70 s^−1^. This is attributed to the shear-induced crystallization.

The flow-induced crystallization has been studied in depth [37,38,39,40,41,42,43,44,45]. It occurs when the Rouse–Weissenberg number *Wi_R_* is above unity as follows:

(4) $$Wi_{R}\equiv \dot{\gamma }\tau_{R}>1$$

where *τ_R_* is the Rouse time.

In this experiment, the high-molecular-weight fraction in PLA must satisfy the relationship, *Wi_R_* > 1, at a shear rate of 70 s^−1^.

**Figure 9:**
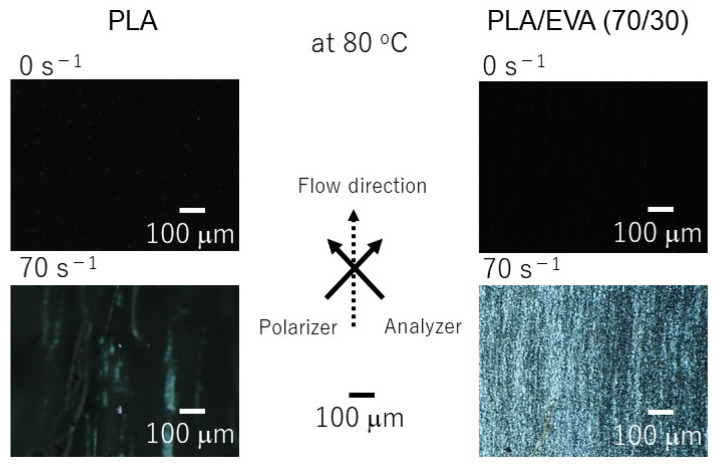
POM images of PLA and PLA/EVA (70/30) under crossed polarizers at 80 °C during cooling.

PLA/EVA showed shear-induced crystallization even at 50 s^−1^. Moreover, the light intensity of the sample with a shear history at 70 s^−1^ was high compared with that of pure PLA. These results demonstrate that the addition of EVA enhanced the shear-induced crystallization of PLA. The corresponding POM images provide a visual confirmation of the shear-induced crystallization, as shown in Figure 9. PLA/EVA (70/30), after exposure to shear, showed bright stripes over the whole area, indicating a high degree of crystallinity with chain orientation. Similar behavior was also detected for PP/LDPE blends, in which LDPE was in the dispersed phase. During cooling, the long-chain branched polymer showed strain hardening even in shear flow at a constant shear rate because *Wi_R_* increased with decreasing temperature [46]. Such behavior is suggested by previous reports on the transient shear stress under exponential shear flow [47,48,49,50,51,52]. Excess deformation occurred in the continuous phase as a result of the exponential increase in *Wi_R_* during cooling at a constant cooling rate. The same was observed in this study, which resulted in the pronounced shear-induced crystallization of PLA.

The result indicates accelerated crystallization even for T-die extrusion. However, in this experiment, PLA crystallization was not detected in the extruded films, although the chain orientation was developed. Presumably, the work by shear flow in the die, which is also important for flow-induced crystallization [39,41,44,45], and/or the crystallization period, which is determined by the time attached to the chill roll, i.e., residence time in the air gap, were insufficient to show PLA crystallization.

## 4. Conclusions

The impact of EVA addition on the rheological properties in the molten state, crystallization behavior after shear flow, and processability at T-die extrusion were studied using a conventional PLA. EVA was immiscible with PLA, and the blend showed a sea–island structure when the EVA content was 30 wt%. Under high shear stress conditions, shear viscosity was greatly reduced by the EVA provided, presumably owing to the interfacial slippage between PLA and EVA. In contrast, strain hardening in the transient elongational viscosity was introduced by the addition of EVA, which was responsible for the reduction of the neck-in level in T-die extrusion. During the elongational flow, deformed EVA droplets were negligibly deformed beyond a critical strain because of the strain hardening of EVA, leading to excess deformation of PLA. Therefore, the chain orientation of PLA was detected in the extruded sheet for PLA/EVA. Finally, shear-induced crystallization was found to be accelerated by the EVA addition. Although PLA crystallization was not detected for the T-die extrusion performed in this study, such characteristics are expected to contribute to improving the processability of PLA in certain operations.

## Figures and Tables

**Table 1:** Enthalpies of cold crystallization and melting at the first heating and that of crystallization at the slow cooling (2 °C min^−1^).

Samples	Cold Crystallization of PLA at the First Heating (J g^−1^)	Melting of PLA at the First Heating (J g^−1^)	Crystallization of PLA at the Cooling 2 °C min^−1^ (J g^−1^)
PLA	9.3	10.6	1.1
PLA/EVA (70/30)	6.5	7.8	0.8

**Table 2:** Film width and birefringence of extruded films.

	Width (mm)	Δ*n* × 10^4^
PLA	28.2	1.1
PLA/EVA (70/30)	30.0	13.8

## Data Availability

The original contributions presented in this study are included in the article. Further inquiries can be directed to the corresponding author.
